# Chronic ulcerative stomatitis: A systematic review of the clinical and microscopic features

**DOI:** 10.4317/medoral.22213

**Published:** 2019-10-27

**Authors:** Túlio Morandin Ferrisse, Daphine Caxias Travassos, Audrey Foster Lefort Rocha, Elaine Maria Sgavioli Massucato, Andreia Bufalino

**Affiliations:** 1 Oral Medicine, Department of Diagnosis and Surgery, São Paulo State University (Unesp), School of Dentistry, Araraquara, São Paulo, Brazil

## Abstract

**Background:**

the purpose of this study was to perform a systematic review regarding clinical and histopathological characteristics, immunopathological findings, and treatment for chronic ulcerative stomatitis (CUS).

**Material and Methods:**

articles in English, published from January 1962 up to November 2017, assessing clinical and immunological features, treatment, and follow-up of patientes with CUS, were retrieved from three databases (PubMed, Cochrane Library and SCOPUS). A manual literature search was also conducted. A total of 12 studies met inclusion criteria, therefore, were analyzed in this review.

**Results:**

CUS shares similiar clinical and microscopic features to those found in oral lichen planus (OLP) and oral lichenoid lesions (OLL). Hence, direct immunofluorescence (DIF) is indispensable to define a final diagnosis. Due to the poor sample availability in the current literature, it is not possible to accurately confirm the prevalence and features of CUS.

**Conclusions:**

in order to better evaluate this condition’s findings, further studies with a greater amount of similar immune-mediated diseases should be performed.

** Key words:**Chronic ulcerative stomatitis, immune-mediated diseases, immunofluorescence, lichen planus.

## Introduction

Chronic ulcerative stomatitis (CUS) is a poorly understood chronic condition that causes painful, exacerbating, and remitting ulcerations, particularly in oral mucous membranes (1). To the best of our knowledge, there are only few cases of CUS reported in the English-language literature. Since this condition may be confounded with other autoimmune diseases, especially oral lichen planus (OLP), it is likely that many cases are misdiagnosed (2,3). Histopathologic findings are non-specific. However, suggestive features include atrophic, parakeratinized and stratified squamous epithelium, lichenoid inflammatory cell infiltrates, basal cell degeneration, and cytoid bodies (1). Moreover, direct immunofluorescence (DIF) of lesional and perilesional specimens shows the presence of autoantibodies with a stratified epithelial specific-antinuclear antibody (SES-ANA) pattern (4). These autoantibodies target an antigen, deltaNp63alpha, which is a nuclear protein normally present in the basal and parabasal cells of stratified squamous epithelia (5,6).

Distinctively from other immunologically mediated conditions, such as OLP, mucous membrane pemphigoid, pemphigus vulgaris, linear IgA disease, and lichenoid drug reaction (6,7), CUS does not show a good response to corticosteroids when compared to other treatments, as hydroxychloroquine (2). Hence, an accurate diagnosis is extremely important to establish an appropriate management (8).

Currently, no study has systematically evaluated the clinical and histopathological characteristics and the immunofluorescence pattern of CUS. Thus, the aim of the present study is to provide an overview of the above-mentioned features observed in CUS. This research followed the Preferred Reporting Items for Systematic Reviews and Meta-Analyses (PRISMA) checklist (9). The PRISMA statement consists of a 27-item checklist and a four-phase flow diagram.

## Material and Methods

- Eligibility criteria

The research question was based on the “PVO” strategy for systematic exploratory review. *P* stands for the population, context and/or problem situation, V for the variables, and O for the desirable or undesirable outcomes. This study aimed to answer the following focused question: Do clinical, histopathological, and immunopathological features of CUS overlap with other autoimmune disorders characteristics?

Inclusion criteria for our systematic review were (i) studies describing clinical, histopathological, and immunopathological findings in oral chronic ulcerative stomatitis patients; (ii) cases reports, case series and cross-sectional studies; and (iii) articles published in English.

Criteria for excluding studies were (i) experimental analysis conducted in animals or *in vitro* models; (ii) reviews articles, letters, personal opinions, book chapters, or conference abstracts; (iii) articles published by the same authors or groups with duplicate patient data; and (iv) studies in which patients had associated systemic disorders (e.g., Sjögren’s syndrome and systemic lupus erythematosus).

- Search strategy

Two independent examiners conducted an electronic search in the PubMed/MEDLINE, Cochrane Library and SCOPUS databases for articles published between January 1962 and November 2017.

The following search terms and combinations were used: “oral chronic ulcerative stomatitis” and “chronic ulcerative stomatitis”. In addition, a handsearching was conducted through the journals Oral Diseases, Head & Neck Pathology, International Journal of Maxillofacial Surgery, Journal of Dental Research, and Oral Surgery, Oral Medicine, Oral Pathology, Oral Radiology.

Based on the studies titles and abstracts, two independent researchers selected and classified the articles as included or excluded in the review. The Mendeley Reference Manager Software was used to delete duplicate articles. Data was extracted from the selected articles, and an independent researcher guided the development of this review. Studies were analyzed and discussed. Any possible disagreements during the process were solved before proceeding to the next steps.

- Data extraction and analysis

The following data was extracted from the studies: (a) demographic data (age, gender and ethnicity); (b) number of investigated patients; (c) detailed clinical descriptions of oral lesions (oral sites, signs and symptoms); (d) description of histophatological characteristics; (e) description of immunopathological characteristics; (f) treatment choice, and (g) follow-up duration. The Level of Evidence (LoE) for each study was determined according to the guidelines of the Oxford University Centre for Evidence-based Medicine (10).

## Results

- Search results

**Figure 1:**
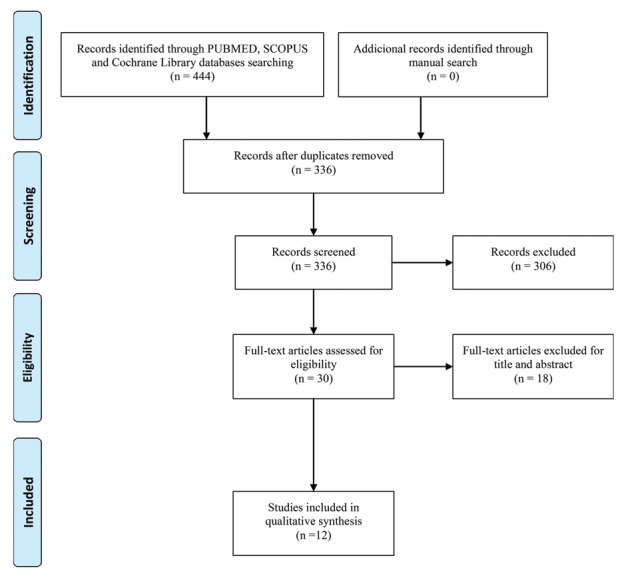
Flow diagram for study selection; 12 studies were identified for analysis (as adapted from the PRISMA statement).

The article selection process is summarized in the flow diagram presented in Fig. 1. Initial electronic search yielded 444 articles. 108 duplicate articles were excluded, therefore, 336 papers remained in the study.

After title and abstract screening, 306 articles were excluded. A total of 30 articles were eligible for full-text evaluation. Subsequently to full-text evaluation, 12 articles were included for qualitative analysis (1,2,4,11-19). Two studies were a cross-sectional, five were case-series and five were case reports. Inter-examiner agreement test (kappa statistic) was applied. For the PubMed/Medline, Cochrane Library and SCOPUS databases, assessment was 100% (κ = 1). For handsearching, the test was also 100% (κ = 1). Characteristics of included studies and their LoE are summarized in Table 1.

**Table 1:**
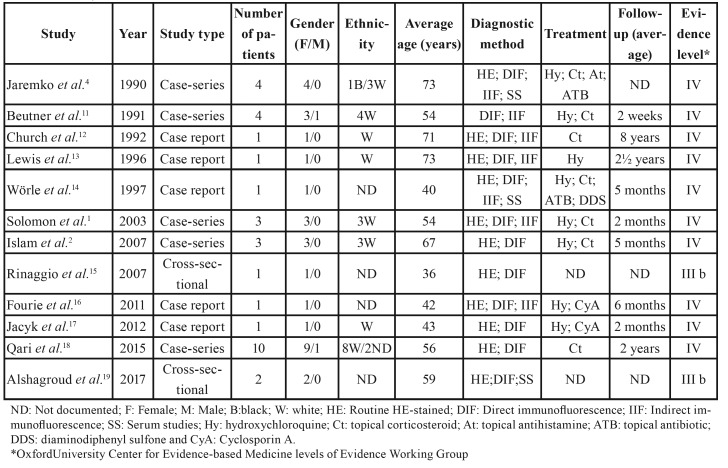
Summary of the 12 included studies characteristics.

Selected studies reported 32 patients with CUS. Four articles were excluded from the systematic review owing to the lack of sufficient details regarding clinical presentation, microscopic features or treatment in relation to individual subjects (3,7,8,20). Additionally, three patients were excluded, as they presented Sjögren’s syndrome (2,6) or systemic lupus erythematosus (19) associated with CUS.

- Synthesis of results

The systematic review showed that CUS affects patients at an average age of 57 years (range 36-81 years), with a strong female predilection (30 women and 2 men). The majority of studies have European origin; consequently, 75% of CUS patients were white. Simultaneous oral and skin lesions were observed in 18.7% of the cases. Involvement of multiple oral sites was reported in nearly 78% cases, mostly affecting areas that included buccal mucosa, tongue, and gingiva. Bilateral presentation was observed in four cases. The condition generally presents as a symptomatic erosive or ulcerative lesion with subtle white reticular striations. Nonetheless, a pure erosive and ulcerative stomatitis may occur. Patients usually suffer from varying degrees of discomfort including pain, dry mouth, and irritation. A summary of the clinical findings obtained in the included studies is shown in Table 2.

**Table 2:**
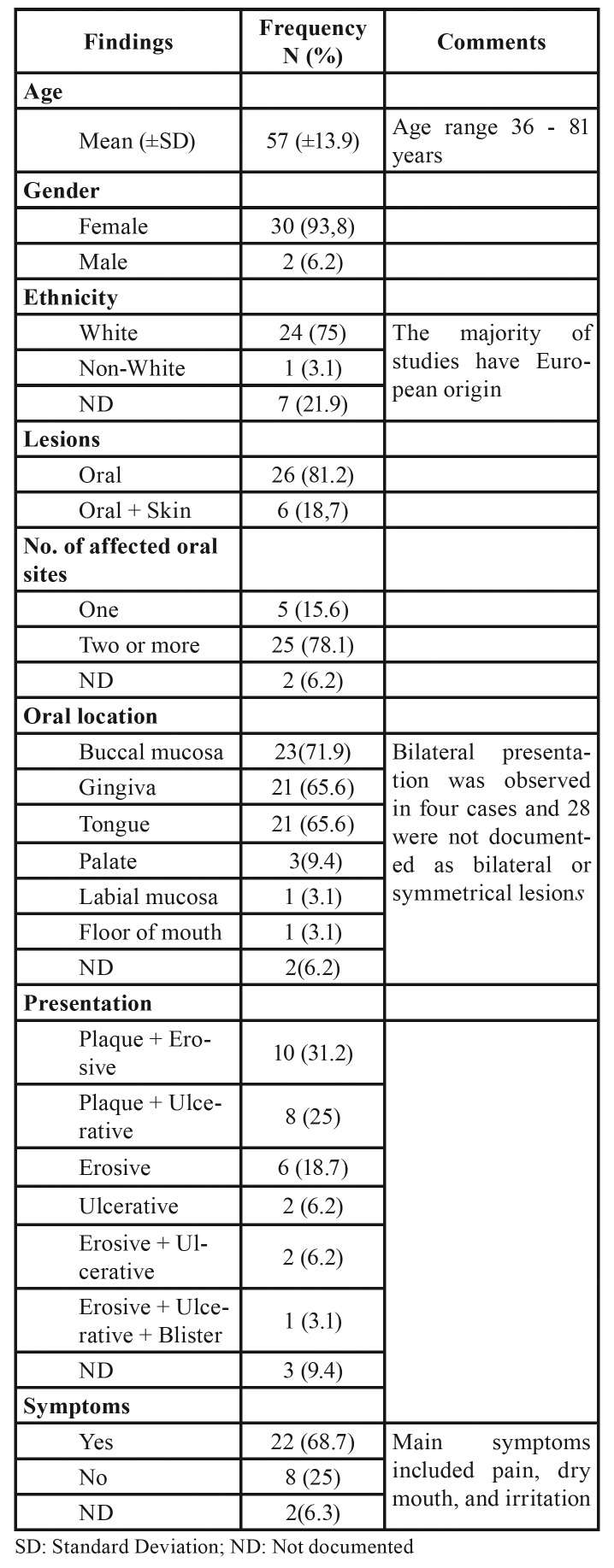
Summary of clinical findings in 32 cases.

Light microscopic features in some CUS cases are reported to be indistinguishable from OLP or to show oral lichenoid lesions (OLL) features, sometimes it is also described as a nonspecific ulcer. In our systematic review, according to a set of modified World Health Organization (WHO)’s diagnostic criteria for OLP and OLF (21), 13 cases met the microscopic criteria of OLP, while 17 cases displayed as OLL. In all cases, histopathological diagnosis of CUS was reached through DIF. DIF in lesional and perilesional oral mucosal specimens revealed a finely speckled granular pattern of IgG deposition, which is normally expressed in basal cell nuclei of stratified squamous epithelia. In addition, we observed that 18 cases (56%) showed a lichenoid fibrin deposits pattern. Moreover, the DIF for anti-human IgA, IgM and C3 were only positive in 20%, 0% and 0% cases of CUS, respectively. The DIF outcomes for the 32 evaluated patients are summarized in Table 3.

Hydroxychloroquine was found to be the drug of choice in limiting the disease. Patients remained asymptomatic for a long time after its usage. Most reports state that in spite of the discontinuation of hydroxychloroquine therapy, the majority of patients stayed in remission or had a disease-free status. Dose adjustments relative to body weight, severity, and extent of involvement are of great importance, although there is a recommended starting daily dose of 200 mg, to a maximum of 800 mg (2,22).

## Discussion

CUS was first reported as a unique entity with excellent response to hydroxychloroquine (4). This entity has been known as a chronic ulcerative lesion associated with a stratified epithelium-specific antinuclear antibody (SES-ANA) that reacts predominantly with the epithelium’s basal layer (4). Many groups have described cases or series-reports of oral chronical ulcerations lesions with SES-ANA immunologic pattern (1-4,6,7,11-20). However, the small number of reports does not allow the establishment of the real prevalence and features of CUS. This is probably related to many cases that are actually undiagnosed. To the best of our knowledge, the present systematic review is the first to assess the clinical, microscopic and immunological characteristics, and therapeutic outcomes systematically.

The present results indicate that CUS is most common in white women in the fifth decade of life. The exclusive oral cavity involvement occurred in 81.2% of the cases. Nevertheless, skin or other mucous membranes may be involved as well. The condition has been reported as progressive painful erythematous gingival lesions, with large, tender erosions, ulcerations, and vesicle formation, raising a clinical suspicion of erosive lichen planus. Here, we show that CUS often presents as a symptomatic erosive or ulcerative lesion with subtle white reticular striae. Main symptoms include pain, dry mouth, and irritation. Other symptoms are nervousness, fatigue, inability to eat, inability to drink hot or cold drinks, weight loss, and sleeplessness (1,2,4,11-14,16).

**Table 3:**
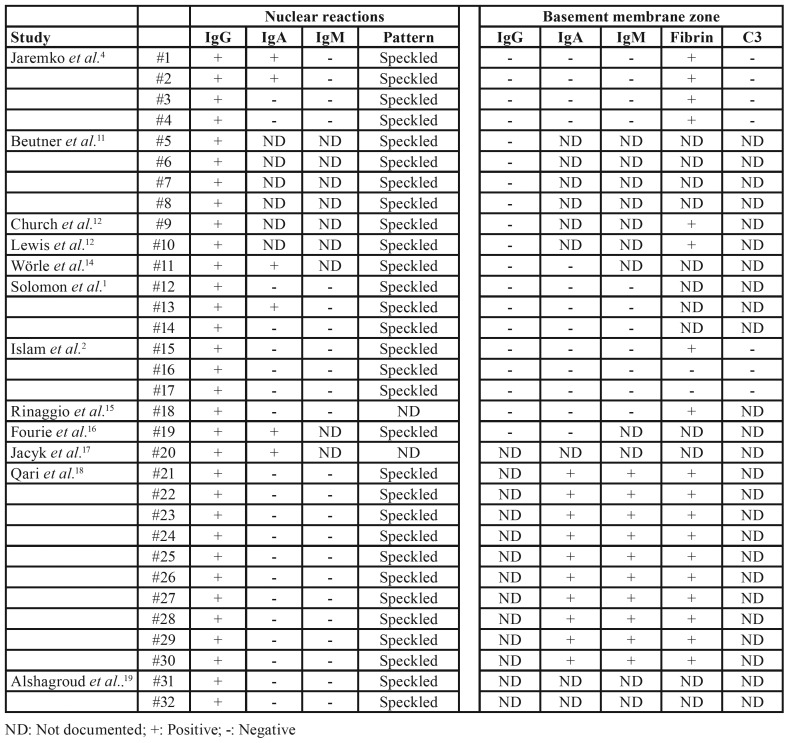
Summary of immunofluorescence findings in 32 cases.

CUS often shares some clinical and histopathological features with OLP, lichenoid stomatitis, mucous membrane pemphigoid, dermatitis herpetiformis, linear IgA disease, pemphigus vulgaris, erythema multiforme, pyostomatitis vegetans, and epidermolysis bullosa acquisita (7). Our results showed that DIF in lesional and perilesional oral mucosal specimens is indispensable to establish the differential diagnosis of CUS. The finely speckled granular pattern of IgG deposition in basal cell nuclei of stratified squamous epithelia and a lichenoid fibrin deposits pattern were the most accurate and reliable features to address the lesion as being CUS. Nonetheless, experienced laboratory technicians are mandatory to conduct DIF, a high cost test. Otherwise limiting its performance. As a result, many oral ulcerative conditions, including CUS, are empirically treated without an accurate diagnosis (2).

Recently, a group proposed the development of Enzyme-linked Immunosorbent Assay (ELISA) for the detection of IgG antibodies to the N-terminal immunogenic portion of DNp63a (DNp63) in a cohort of CUS sera (8). However, the lack of a greater number of positive controls was an important limitation in this study. Therefore, future studies are needed to better understand these findings.

The results of this systematic review showed that severe cases of CUS seem to be controlled with the use of hydroxychloroquine sulfate (1,2,4,11,13,14,16,17,22). Important to notice, hydroxychloroquine must be carefully administered due to its side effects, such as retinopathy, toxic psychosis, neuropathy, agranulocytosis, and aplastic anemia, which may lead to treatment discontinuation (13). Neuromuscular and hematologic complications are usually reversible, whereas retinal are not. In this manner, close follow-up of patients who take hydroxychloroquine is required (12). Alternatively, chloroquanidine, which has a high therapeutic index and appears to be well tolerated, may become the drug of choice to treat this condition. Furthermore, less severe cases can be controlled by selected topical corticosteroids, although lesions tend to recur (1,2,4,11,12,14,18).

Interestingly, in an analysis of 42 OLP cases, derived from a previous published single-center, randomized, controlled, single-blind study, which had different responses to two types of treatment (topical corticosteroid or laser phototherapy), high levels of acetyl-histone H3 at lys9 (H3K9ac) were intrinsic related to failure in responding to the proposed treatment or to disease recurrence shortly after therapy. Furthermore, presence of DNA double-strand breaks (DSBs), demonstrated by accumulation of H2AX histones, were also found in those highlighted cases, indicating genomic instability and poor response to treatment (23). Since OLP is similar to other oral immunological disorders, as CUS, it would be possible that these histones modifications, if found in CUS cases, could explain the disorder’s inadequate response to corticosteroid therapy.

Conclusion

In the present review, only 12 studies retrieved from the search met the inclusion criteria and were evaluated. However, these studies presented significant limitations as showed by the LoE analysis. Considering this, further studies with a larger number of OLP and OLL should be performed to reliably assess the clinical, microscopic and immunological characteristics of CUS. DIF is obligatory in oral tissue biopsies from patients with a medical history of recurrent erosive or ulcerative oral lesions present for an extended period without an adequate corticosteroid therapy response. Additionally, our group suggests new studies regarding the role of H3K9ac and H2AX histones in CUS cases in order to evaluate if there is a relation among high levels of both histones, the condition’s behavior, and poor outcomes after corticosteroid treatment.
